# Planktonic ciliate communities along an environmental gradient in the Nile Delta (Damietta region, Egypt)

**DOI:** 10.1038/s41598-024-69551-9

**Published:** 2024-08-28

**Authors:** Wael S. El-Tohamy, Russell R. Hopcroft

**Affiliations:** 1 Zoology Department, Faculty of Science, Damietta University, Damietta, Egypt; 2 College of Fisheries and Ocean Sciences, University of Alaska Fairbanks, Fairbanks, AK USA

**Keywords:** Planktonic ciliates, Spatial distribution, Environmental stress, Bioindicator, Damietta region, Ecology, Zoology, Environmental sciences

## Abstract

The spatial patterns of planktonic ciliate communities were studied from May to June 2019 in the Nile Delta’s Damietta region, southeastern Mediterranean. The ciliate communities were sampled from twenty-five sites of five stressed domains with spatial gradients of environmental status. A total of 32 ciliate taxa with six dominant species were identified, comprising 21 tintinnids and 11 aloricate ciliates. The abundance and richness of each ciliate group varied geographically and were most strongly influenced by salinity variations; tintinnid ciliates attained high abundance and richness at high salinity sites in the harbour and coastal region and decreased within the estuary upstream. Aloricate ciliates were poorly represented at most sites but were a substantial proportion of upstream estuarine sites. Multivariate/univariate analyses demonstrated that spatial patterns of the ciliate communities were significantly correlated with environmental variables, especially salinity, chlorophyll-a, and nutrients, either alone or in combination with one another. These results indicate that the ciliates can be useful bioindicators in stressed environments while also allowing the detection of impacts on short time scales by rapidly responding to environmental variations.

## Introduction

Planktonic ciliates are common members of unicellular eukaryotic organisms in marine ecosystems^1^. They are characterized by high species diversity and often dominate the microzooplankton community^2^. Planktonic ciliates also act as mediators by grazing on nanoplankton while serving as prey for metazoans^3^. With their short generation times and delicate pellicles, they may respond more quickly to environmental changes than metazoans^4^. Numerous ciliates can also tolerate extreme environmental conditions compared to macrofauna^5,6^. Thus, ciliated protozoa have been successfully used as a bioindicator for assessing water quality status at both species and community levels in aquatic ecosystems^7–9^.

Many studies on the diversity, spatial, and temporal patterns of planktonic protozoa have been conducted in Egyptian waters. For instance, studies have focused on the Mediterranean coast^10,11^, Manzala Lake^12^, Burullus Lake^9^, and throughout the Nile River^13–15^. Despite all these efforts, we still need to know several fundamental aspects regarding the diversity and spatial patterns of a prominent component of the planktonic protozoa, the planktonic ciliates^16^. Although many studies have focused on ciliate's relationships to marine water quality, more information on the environmental factors governing them is still needed. The Nile Delta region may be a useful location to explore such associations.

The selected study area is a part of the northern Nile Delta in the southeastern Mediterranean. It covers an area of about 35 km^2^ of four different locations: Damietta Harbour, Barge Canal, Damietta Nile estuary, and the Damietta Sea coast. In recent decades, the northern Nile Delta has been increasingly impacted by anthropogenic activities (e.g., industry, agriculture, and aquaculture); consequently, it is subject to pollution and/or eutrophication events^10,17,18^. Many artificial drainages have become discharge trenches for agricultural runoff, industrial effluents, and urban wastewaters from the Nile Delta region, collectively important sources of pollutants entering the Mediterranean Sea. The principal routes by which the pollutants entered the Delta's coast are the Nile estuary, Damietta Harbour Barge canal, and three large primary drains of Ezbet Setta, Gamasa, and El-Kassara^10^.

Multivariate analyses have proved to be a powerful approach to establishing differences among communities, particularly across spatial scales, and for demonstrating how these patterns vary along gradients of environmental conditions^3,7^. Such approaches provide an efficient means to reduce the complexity inherent in community-based ecological research and facilitate the detection of the environmental parameters that explain this complexity. In this context, the main objectives of this study were: (1) to document the planktonic ciliate community structures at the five domains with contrasting anthropogenic activities; (2) to explore the spatial variations in planktonic ciliates biodiversity; (3) to determine relationships between planktonic ciliate communities and environmental variables.

## Material and methods

### The study area

**Figure 1:**
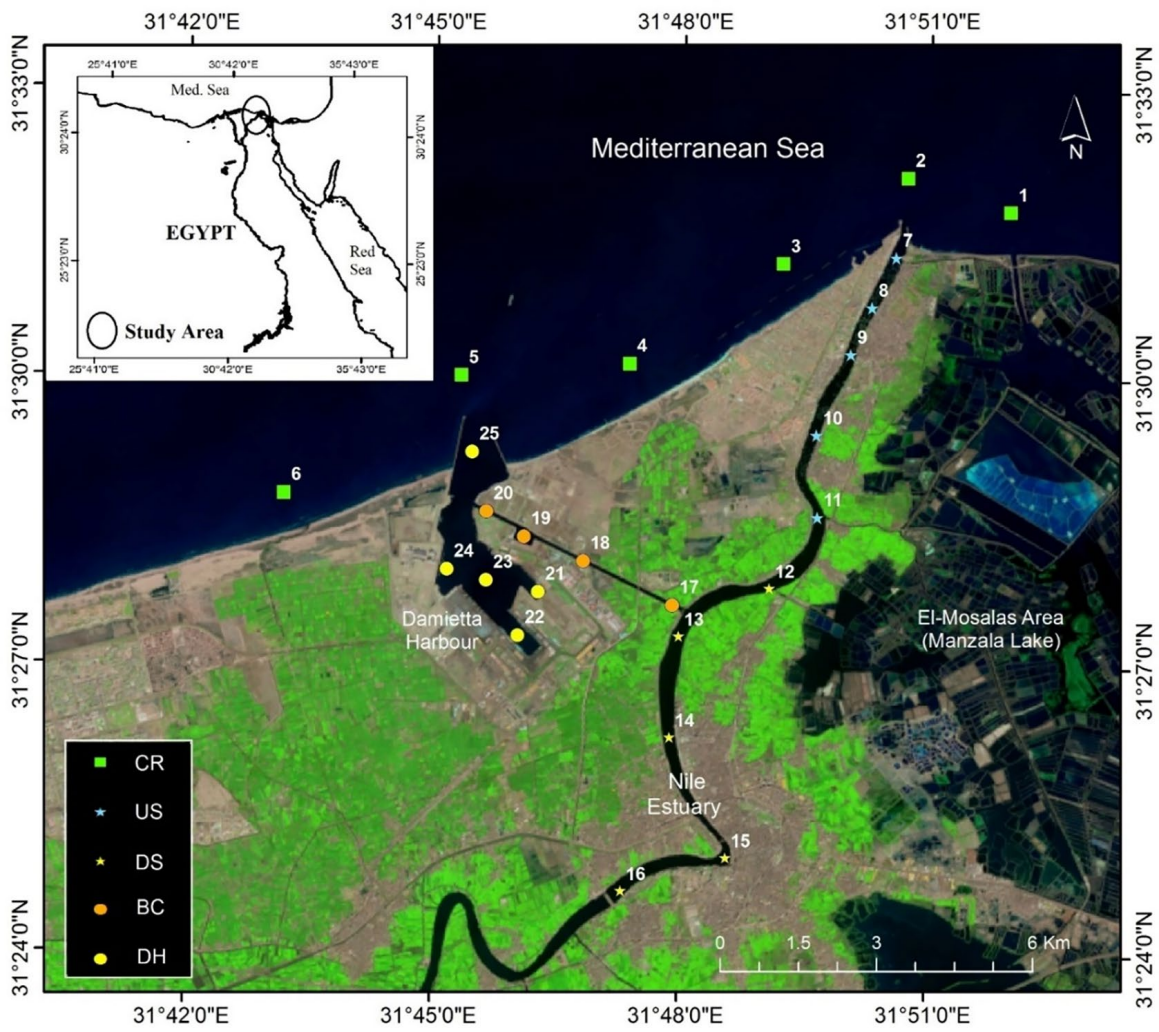
A Map of the study area showing the positions of the sampling sites in Egypt’s Damietta region. This map was generated using Esri ArcMap v. 10.5.

The current study was carried out in 4 regions at the northeastern Nile Delta between 31°20′ N and 31°34′ E, Damietta Harbour with its Barge Canal, Damietta Nile estuary, and the Damietta coast (Fig. 1). The harbour is a semi-closed basin with an area of 3.1 km^2^ and depth range of 5–15 m, connected to the sea through the navigational canal and is linked to the Nile estuary by the Barge Canal that is 4.5 km long and 5–7 m depth. In addition to the direct effects of maritime activities, the harbour water quality could be changed when the shipping materials, such as organic fertilizer and cement, dissolve when they reach the water. The Barge Canal is directly affected by Nile River discharge, sewage, and agricultural effluents. The Damietta Nile estuary's length is about 13 km, and it is completely isolated from the riverine water by the Faraskour dam. Although the dam has six gates, they open randomly and rarely release freshwater into the estuary. Thus, the water properties in the estuary are mainly controlled by the land runoff and the tidal regime^19^. The effluents developed from land runoffs and Manzala Lake canals mostly affect the estuary upstream in contrast to downstream, which is affected more directly by the seawater intrusion from the Mediterranean Sea. Given the limited semidiurnal tidal range (30–60 cm maximum)^20^, typical water movement is sluggish but not completely stagnant. The last sampling region is at the Damietta coast and traverses ~ 15 km with an average depth of 15 m (Fig. 1). The four areas have contrasting anthropogenic impacts. Based on previous studies, the Nile estuary and the Barge Canal were known to be the most heavily stressed areas, with their pollution being mainly in the form of organic pollutants and nutrients from domestic sewage and agricultural and industrial discharges. Damietta Harbour was moderately polluted due mainly to intensive maritime activities and the circulation of waters from the Barge Canal. The coastal region was the least polluted area^17–19^.

### Samples collection and analysis

In this study, 25 sites were selected (Fig. 1): 6 at the coastal region (CR), 5 at the Damietta Harbour (DH), 4 at the Barge Canal (BC), and 10 at the Damietta Nile estuary (5 at the downstream (DS) & 5 at upstream (US)). The samples for analyzing physic-chemical parameters and planktonic ciliates were collected biweekly from the 25 sites mentioned above during May and June 2019. All samples were collected at a depth of 1 m. For quantitative studies and identification of planktonic ciliates, a 2.2-liter Van Dorn sampler was used to filtrate 20 liters of water through a 20-μm mesh plankton net with a mouth diameter of 40 cm^9^. The samples were then concentrated to 50 ml and fixed with acid Lugol’s Iodine solution. Three replicates of 1 ml were transferred to a Sedgwich-Rafter counting tray and then identified and enumerated under an Optika microscope (model: B-130) at 100–200× magnification. Tintinnids were identified using lorica morphology according to Boltovskoy^21^ and Marshall^22^; other ciliates followed Edmondson^23^ and Patterson and Hedley^24^.

Temperature, salinity, pH, and dissolved oxygen concentrations (DO) were measured in situ using a multi-parameter sensor (YSI-Model 85D). Water transparency was measured by using a 25 cm Secchi disc. Samples for nutrient analyses were preserved directly after collection by placing at – 18 °C. Soluble reactive phosphate, ammonium (NH_3_), Nitrite (NO_2_), Nitrate (NO_3_), and silicate (SiO_4_) were later determined according to the manual method of Wetzel and Likens^25^. Chlorophyll-a was measured spectrophotometrically according to the procedure given by Strickland and Parsons^26^.

### Statistical approaches

Mean abundance (Cells l^−1^) was calculated for each species. Traditional biodiversity indices (Shannon-Wiener species diversity, Pielou’s of species evenness, and Margelef species richness) were calculated to explore variations between sampling domains.

Analysis of variance (ANOVA) was used to test for differences in the abiotic and biotic parameters between different domains. The data were tested for normality before analysis and transformed into natural logarithms when necessary to achieve normality. Spearman's correlation was used to describe the relationship between selected parameters. ANOVA and Spearman analysis were performed using SPSS 18. The correlation between the dominant ciliates and environmental factors was analyzed and visualized by the R packages Psych^27^.

Multivariate analyses of spatial variations in planktonic ciliate communities were analyzed using the PRIMER v6.1 package^28^. To explore faunal associations and spatial patterns of communities, we constructed a dendrogram of species using a group-average distribution on Bray–Curtis similarity from square-root-transformed data of each species abundance^29^. The contribution of each species to the average Bray–Curtis similarity among domains was analyzed using the SIMPER (Similarity Percentage Analysis) routine^28^. The spatial differences of ciliate communities among the five sampling domains were summarized using the CAP (canonical analysis of principal coordinates) of PERMANOVA + on Bray–Curtis similarities from the square-root transformed species-abundance data^30^. A vector overlay of Spearman correlations with a coefficient (ρ value) of > 0.2 with the CAP axes was employed to show the influence of the most common species^30^. Differences between groups of samples were tested by the ANOSIM (analysis of similarities)^28^. The relationships among the five sampling domains were also explored using MDS/PCA ordinations. The MDS was constructed based on Bray–Curtis's similarity for square-root transformed species abundance data, while the PCA was based on Euclidean distance from log-transformed environmental data^31^. The biota-environment correlation was tested using the routine RELATE. The submodule BIOENV (biota-environment correlation analysis) explored potential relationships between ciliate community structure and the environmental parameters^28^.

## Results

### Environmental parameters

Environmental parameters showed strong spatial gradients within the study area. Overall, all domains (CR, DH, BC, DS, and US) were significantly different in terms of environmental variables characteristics throughout the study period (PERMANOVA, p < 0.01). All variables showed significant spatial differences except temperature (Table 1). The relatively high values of transparency, ammonia, nitrate, nitrite, and chlorophyll-a (Table 1) indicated the significant influence of anthropogenic activities on these water quality parameters. Along CR and DH, salinity and transparency had higher values than other regions, with a noticeable decrease in the upstream estuary affected by freshwater flow. In contrast, the pH, ammonia, nitrite, and chlorophyll-a values had marked changes, moving to decrease at CR and DH than those of BC, DS, and US, which were relatively similar. DH and BC showed significantly higher concentrations of silicate and soluble reactive phosphate than other sites. The nutrient-rich habitat at the Nile estuary and BC promoted an intensive growth of phytoplankton that could increase water oxygen levels; dissolved oxygen values were significantly higher in those regions when compared to others.

**Table 1:** Environmental variables monitored at the five sampling domains in Egypt’s Damietta region.

Parameters	Sampling sites					ANOVA	
	CR	DH	BC	DS	US	*F*	*P*
T (°C)	24.23 ± 1.90	24.93 ± 1.66	24.06 ± 2.27	23.40 ± 2.66	23.49 ± 2.85	0.74	0.57
pH	8.06 ± 0.07^a^	8.03 ± 0.11^a^	8.35 ± 0.16^c^	8.57 ± 0.39^b^	8.56 ± 0.11^d^	18	˂0.001
Salinity (ppt)	36.44 ± 0.59^a^	36.73 ± 0.92^a^	27.71 ± 4.20^c^	31.58 ± 3.30^b^	23.75 ± 1.30^d^	54.33	˂0.001
WT (cm)	222.50 ± 55.59^a^	198.59 ± 25.74^a^	115.38 ± 26.02^b^	142.20 ± 46.77^b^	136.40 ± 72.14^b^	8.47	˂0.001
DO (mgl^−1^)	5.59 ± 0.82^b^	6.90 ± 0.56^ab^	6.79 ± 0.42^ab^	7.85 ± 2.30^a^	7.41 ± 1.84^a^	4.02	˂0.01
NH_3_ (μgl^−1^)	16.31 ± 11.81^b^	42.60 ± 23.14^ab^	97.46 ± 35.87^a^	60.64 ± 48.55^a^	124.31 ± 197.61^a^	6.85	˂0.01
NO_3_ (μgl^−1^)	23.23 ± 7.37^c^	100.74 ± 68.59^b^	70.24 ± 9.14^b^	69.94 ± 30.26^b^	182.40 ± 97.90^a^	21.19	˂0.001
NO_2_ (μgl^−1^)	13.99 ± 4.53^b^	26.10 ± 21.72^ab^	29.71 ± 7.82^a^	22.18 ± 5.97^ab^	38.31 ± 28.47^a^	3.79	˂0.05
SiO_4_ (μgl^−1^)	59.01 ± 67.31^c^	1045.48 ± 941.77^a^	133.95 ± 76.18^b^	51.89 ± 14.17^c^	109.24 ± 39.59^b^	14.59	˂0.001
SRP (μgl^−1^)	38.96 ± 15.93^bc^	86.82 ± 64.88^ab^	89.93 ± 40.06^a^	47.89 ± 27.18^bc^	29.73 ± 12.22^c^	5.89	˂0.01
Chl-a (μgl^−1^)	3.64 ± 1.35^c^	8.90 ± 2.26^b^	12.61 ± 2.90^ab^	13.00 ± 2.55^ab^	16.41 ± 4.94^a^	42.65	˂0.001

The letters indicate significant differences based on one ANOVA analysis with Tukey’s-b post hoc test.

Wt, water transparency; DO, dissolved oxygen; SRP, soluble reactive phosphate; Chl-a, chlorophyll-a.

### Species distribution and associations

**Figure 2:**
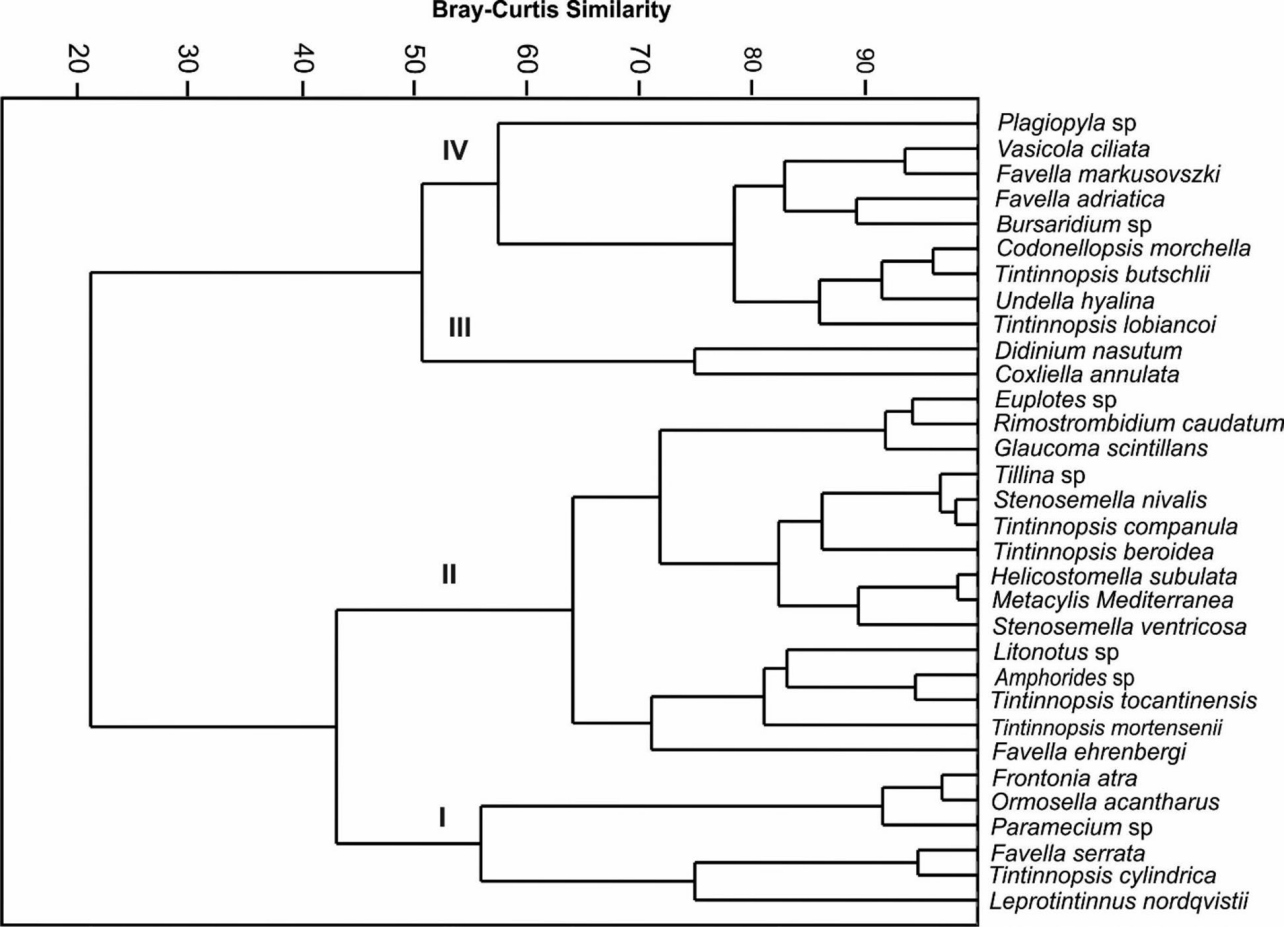
A dendrogram of 32 planktonic ciliates at five domains in Egypt’s Damietta region, plotted using group-average clustering on Bray–Curtis similarities from square root-transformed species abundance data. I–V = groups I–V.

A total of 32 species comprising 11 aloricate (naked) ciliates (belonging to seven different classes) and 21 tintinnids or loricate ciliates (class Spirotricha) were recorded (Table 2). These taxa showed different spatial patterns in species distribution in all domains. Cluster analysis suggested the 32 species fell into four groups (I–IV) at approximately 55% similarity level (Fig. 2): group I was composed of 6 dominant species with high occurrence and contribution, while the other groups (II-IV) represented the assemblages with low occurrence and/or abundance (Fig. 2, Table 2). Three dominant tintinnid forms (e.g., *Favella serrata*, *Leprotintinnus nordqvistii*, and *Tintinnopsis cylindrica*) appeared at all sampling sites, two aloricate forms (e.g., *Frontonia atra* and *Paramecium* sp.) found at the low salinity domains (Barge Canal and Nile estuary), while the tintinnid *Ormosella acantharus* appeared to be correlated mainly with the harbour region and its Barge Canal.

**Table 2:** List of ciliate species recorded at the five sampling domains in Egypt’s Damietta region, including average abundance and occurrence, ranked by the contribution of each species based on the average Bray–Curtis similarity.

Species	CR			DH			BC			DS			US		
	N	F%	%	N	F%	%	N	F%	%	N	F%	%	N	F%	%
Aloricate ciliates															
*Bursaridium* sp.-Col										+	2.60	0.21	+	2.63	0.39
*Didinium nasutum*-Lit										+	1.30	0.20			
*Euplotes* sp.-Spi							+	1.75	0.79				++	4.58	4.97
Frontonia atra*-Olig							+	1.75	0.15	+ +	3.90	15.61	++ +	6.58	21.17
*Glaucoma scintillans*-Olig										+	2.60	2.89	+++	6.58	5.80
*Litonotus* sp. -Lit							+	1.75	0.86	+	2.60	0.58	++	5.26	1.26
*Paramecium* sp*-Olig							++	8.77	1.25	++	2.60	10.13	+++	13.16	46.95
*Plagiopyla* sp.-Pla							+	1.75	0.16	+	1.30	0.05	+	2.63	0.06
*Rimostrombidium caudatum*-Ol													++	5.26	3.69
*Tillina* sp. -Col										++	2.60	3.24	++	10.53	4.27
*Vasicola ciliata*-Pro							+	1.75	0.16				+	5.26	0.45
Tintinnida (Spirotricha)															
*Amphorides* sp.	++	4.50	1.38	+	3.85	0.82	++	1.75	3.82	+	1.30	0.17			
*Codonellopsis morchella*	++	8.11	1.74												
*Coxliella annulata*										+	1.30	0.12			
*Favella adriatica*										+	3.90	0.96			
*Favella ehrenbergii*	++	8.11	3.82	++	7.69	1.90	++	5.14	3.83	++	10.39	6.49	+	2.63	0.08
*Favella markusovszkyi*				++	7.69	0.51									
Favella serrata*	++	4.50	1.90	+++	7.69	6.37	+++	10.53	37.17	+++	12.99	37.47	+++	5.26	8.54
*Helicostomella subulata*	++	8.11	7.43	++	6.41	6.35	++	8.77	5.03	+	3.90	1.28	+		0.03
Leprotintinnus nordqvistii*	+++	10.81	37.48	+++	12.82	9.64	++	14.04	9.26	++	12.99	14.85	++	13.16	0.92
*Metacylis mediterranea*	++	9.91	7.74	+	1.28		++	8.77	8.68	+	1.30	0.64	+	3.95	0.78
Ormosella acantharus*	+	0.90	0.85	+++	6.41	40.63	+++	5.26	19.10						
*Stenosemella nivalis*	+	1.80	0.09	++	6.41	2.58				+	3.90	0.27			
*Stenosemella ventricosa*	++	6.31	3.74	+++	6.41	4.55	+	1.75	0.44	+	5.19	0.85			
*Tintinnopsis beroidea*	+++	3.60	9.92	+	6.41	0.25	++	1.75	3.26	+	2.60	0.32			
*tintinnopsis butschlii*	+	1.80	0.56	++	6.41	0.57				+	1.30	0.03			
*tintinnopsis companula*	++	1.80	1.14	++	6.41	2.66	+	1.75	0.45	+	1.30	0.13			
Tintinnopsis cylindrica*	+++	10.81	12.11	+++	6.41	23.11	++	8.77	2.58	+	6.49	0.57	+	3.95	0.12
*Tintinnopsis lobiancoi*	++	5.41	2.15	+	6.41	0.08	+	1.75	0.08	++	1.30	0.39			
*Tintinnopsis mortensenii*	++	6.31	2.84				+	3.51	0.26	+	3.90	0.72	+	3.95	0.36
*Tintinnopsis tocantinensis*	++	6.31	4.20				++	5.26	2.44	+	1.30	0.12	+	2.63	0.16
*Undella hyalina*				+	1.28		+	1.75	0.13	+	5.19	1.62			

Abundance, N (Cells l^−1^): + 1–9, ++ 10–99, +++  > 100; Frequency of occurrence, F%; Species contribution, %

Classes abbreviations of aloricate ciliates: Col, Colpodea; Lit; Litostomatea; Spi, Spirotricha; Olig, Oligohymenophorea; Pla, Plagiopyla; Ol, Oligotrichea; Pro, Prostomatea.

### Spatial patterns of protozoan communities

**Figure 3:**
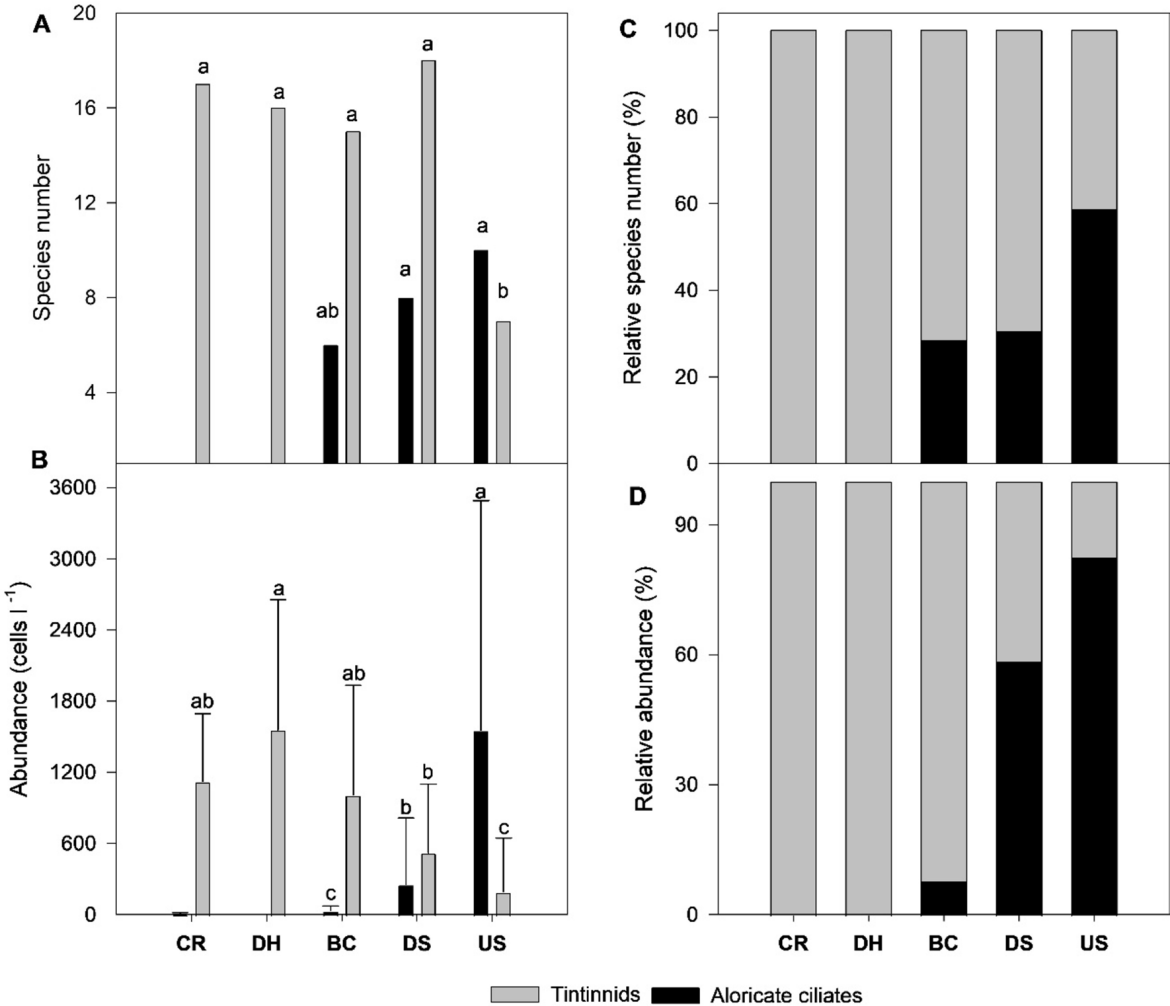
Spatial variations in species number (**A**), abundance (**B**), relative species number (**C**), and relative abundance (**D**) of planktonic ciliates from the five domains in Egypt’s Damietta region. The letters indicate significant differences based on one-way ANOVA with the Tuckey’s test.

The ciliate communities at the five sampling domains showed clear spatial differences in both species’ composition and abundance (Fig. 3). Species numbers showed a maximum mean value at the DS domain and a minimum at the DH domain. However, the mean abundance was highest at the US domain (mean 1745.7 ± 2109.8 cells l^−1^) and lowest at the DS (mean 776.3 ± 741.4 cells l^−1^) domain. In terms of both relative species number and relative abundance, three structural ciliate communities can be identified: (1) those absolutely dominated by tintinnids (e.g., CR and DH domains), (2) those dominated by tintinnids and aloricate ciliates with tintinnids being the primary contributor (e.g., BC and DS domains), and (3) those dominated by aloricate ciliates and tintinnids with aloricate ciliates the greatest contributor (e.g., US domain) (Fig. 3). It should be noted that these spatial patterns of ciliate communities were consistent with the pattern previously described in the water quality variations between the sampling sites (Table 1, Fig. 3).

**Figure 4:**
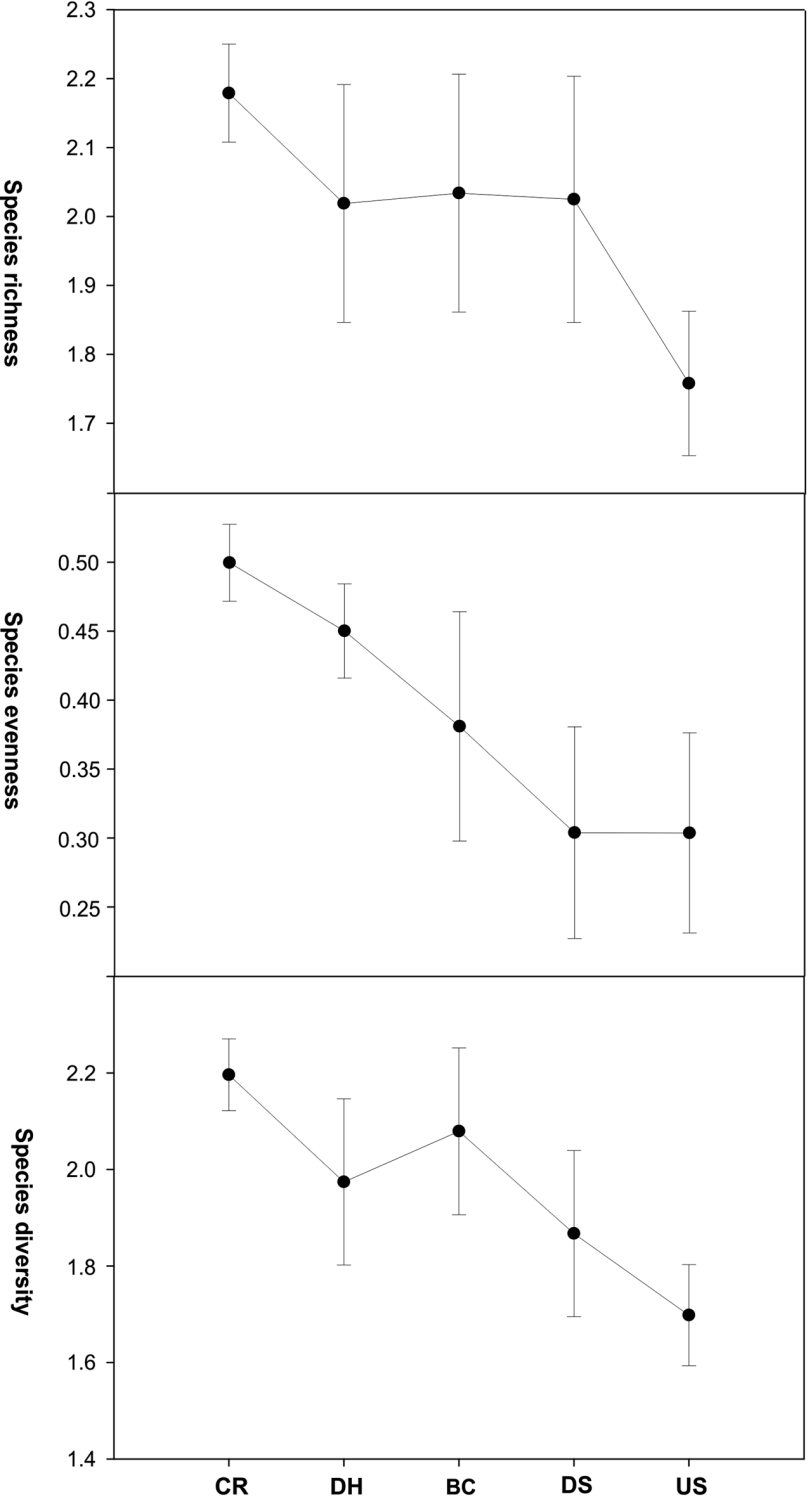
Traditional biodiversity measures species richness, species evenness, and species diversity of planktonic ciliate communities demonstrating variable patterns among five sampling domains in the Damietta region.

Traditional biodiversity indices showed similar dynamics (Fig. 4), i.e., generally increased from the CR to the BC, followed by a decrease in the Nile estuary. Variations in the biodiversity of ciliate fauna may reflect the suitable environmental conditions at the coastal region compared with others.

**Figure 5:**
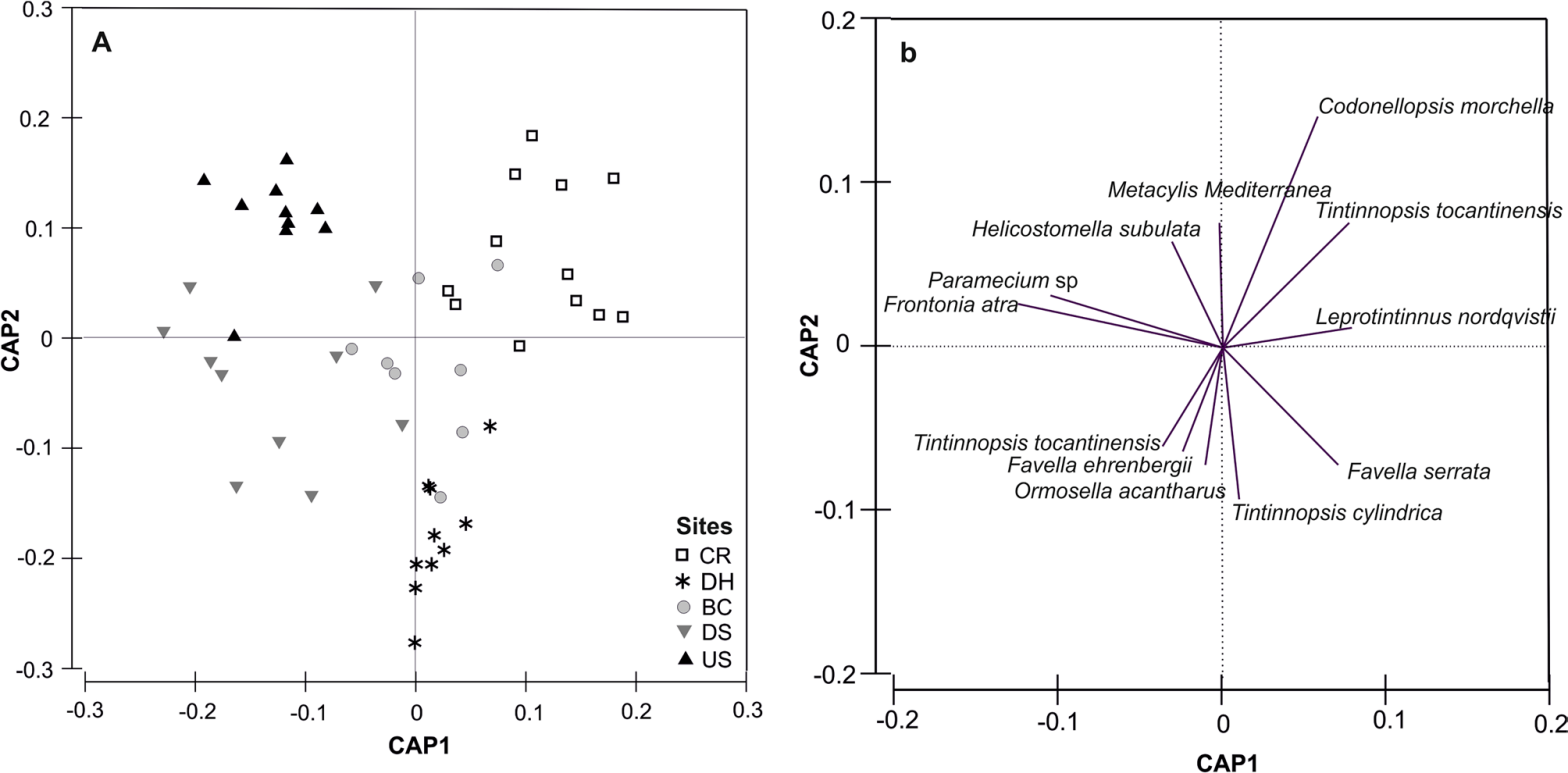
Canonical analysis of principal coordinates (CAP) on Bray–Curtis similarities from square root -transformed species-abundance data of five sampling domains in the Damietta region during the period from May to June 2019 (**A**) and correlations of 12 common species that showed the highest correlations with the two CAP axes (**b**).

The CAP ordination showed a spatial pattern of ciliate communities (Fig. 5A). The first canonical axis separated the ciliate communities sampled at domains CR and DH (on the right) from those at the other three domains (mostly on the left), while the second canonical axis discriminated US and CR samples (Upper) from those at other (lower). ANOSIM test revealed that there were significant differences among the five domains (global R = 0.47, P < 0.001) and between each pair of domains (P < 0.01), apart from domains pairwise test, BC-DH and DS-BC were non-significant (R = 0.05, P = 0.193 and R = 0.15, P > 0.05, respectively).

A vector overlay of correlations to common species showed that of the top six ranked contributors at sampling domains, vectors for three tintinnid species, namely *Codonellopsis morchella*, *Leprotintinnus nordqvistii*, and *Tintinnopsis tocantinensis*, pointed toward the sample cloud of CR (upper right), another three tintinnids, *Favella serrata*, *Ormosella acantharus,* and *Tintinnopsis cylindrica* toward that of DH (lower right), two aloricate ciliates, *Frontonia atra* and *Paramecium* sp toward that of US (upper left), and the remaining four toward those of the other two domains (Fig. 5b).

### Linkage between planktonic ciliate biodiversity and abiotic factors

**Figure 6:**
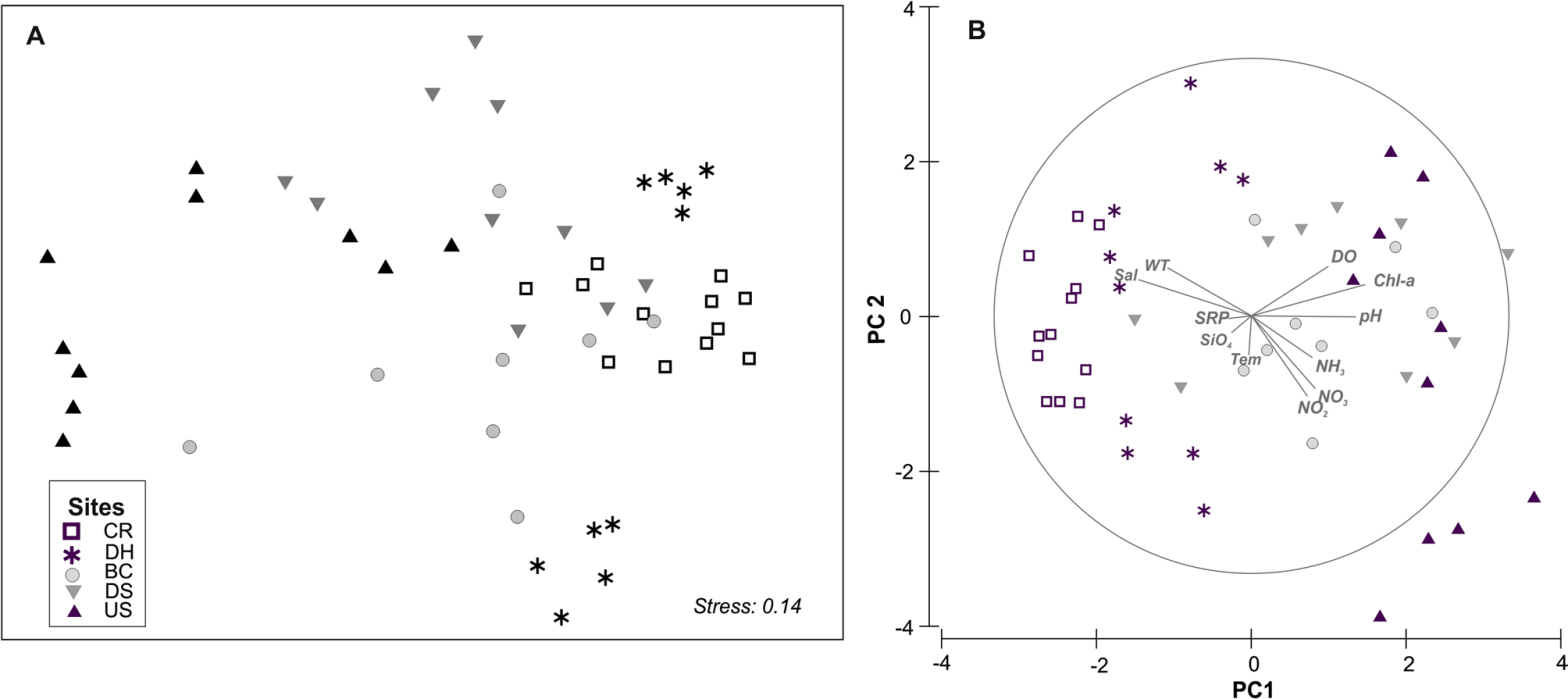
Multidimensional scaling (MDS) for spatial patterns of planktonic ciliate communities (**A**) on Bray–Curtis similarities for square root abundance data, and principal component analysis (PCA) ordination (**B**) based on log-transformed abiotic data of five sampling domains in Damietta region. Tem, temperature; Sal, salinity; WT, water transparency; SRP, soluble reactive phosphate; Chl-a, chlorophyll-a.

The MDS/PCA multivariate approaches revealed that the spatial patterns of the ciliate communities were consistent with those of the environmental parameters (Fig. 6). In both cases, there were high similarities between the three most stressed domains (DS, US, and BC), and somewhat lower similarity between the moderately and least polluted sites (DH and CR). The RELATE analysis revealed a significant correlation between the spatial patterns of planktonic ciliate communities in abundance and changes in environmental variables (R = 0.369; P = 0.001). For PCA, the two principal components explained 55.4% of the total spatial environmental variability. It should be noted that salinity and chlorophyll-a were strongly associated with PCA 1, which was the primary contributor to the spatial environmental patterns (36.5%). For the five domains, the correlations between ciliate abundances and environmental parameters were established by the BIOENV analysis. The results showed that the spatial variations in ciliate communities correlated with the environmental variables, especially temperature, salinity, transparency and chlorophyll-a, either alone or in combination with one another. It was also noted that salinity and chlorophyll-a were the only variables included in most correlations (Table 3), indicating that these were the key factors related to ciliate distribution in the study area. The previous findings showed that the best matching with the spatial variations in ciliate community structure occurred with salinity variations and food availability.

**Table 3:** Summary of Biota-environment (BIOENV) analysis results, with the 10 best correlations corresponding to different variables in the Damietta region.

Rank	R-Value	Environmental variables
1	0.357	Temperature, Salinity, Chlorophyll-a
2	0.403	Temperature, Salinity, Water transparency, Chlorophyll-a
3	0.287	Temperature, Salinity
4	0.340	Salinity, Water transparency, Chlorophyll-a
5	0.286	Salinity, Chlorophyll-a
6	0.332	Temperature, Salinity, NO_3_, Chlorophyll-a
7	0.381	Temperature, Salinity, SiO_4_, Chlorophyll-a
8	0.381	pH, Salinity, Chlorophyll-a
9	0.380	Salinity, NO_3_, Chlorophyll-a
10	0.295	Salinity

R-value: Spearman correlation coefficient.

**Figure 7:**
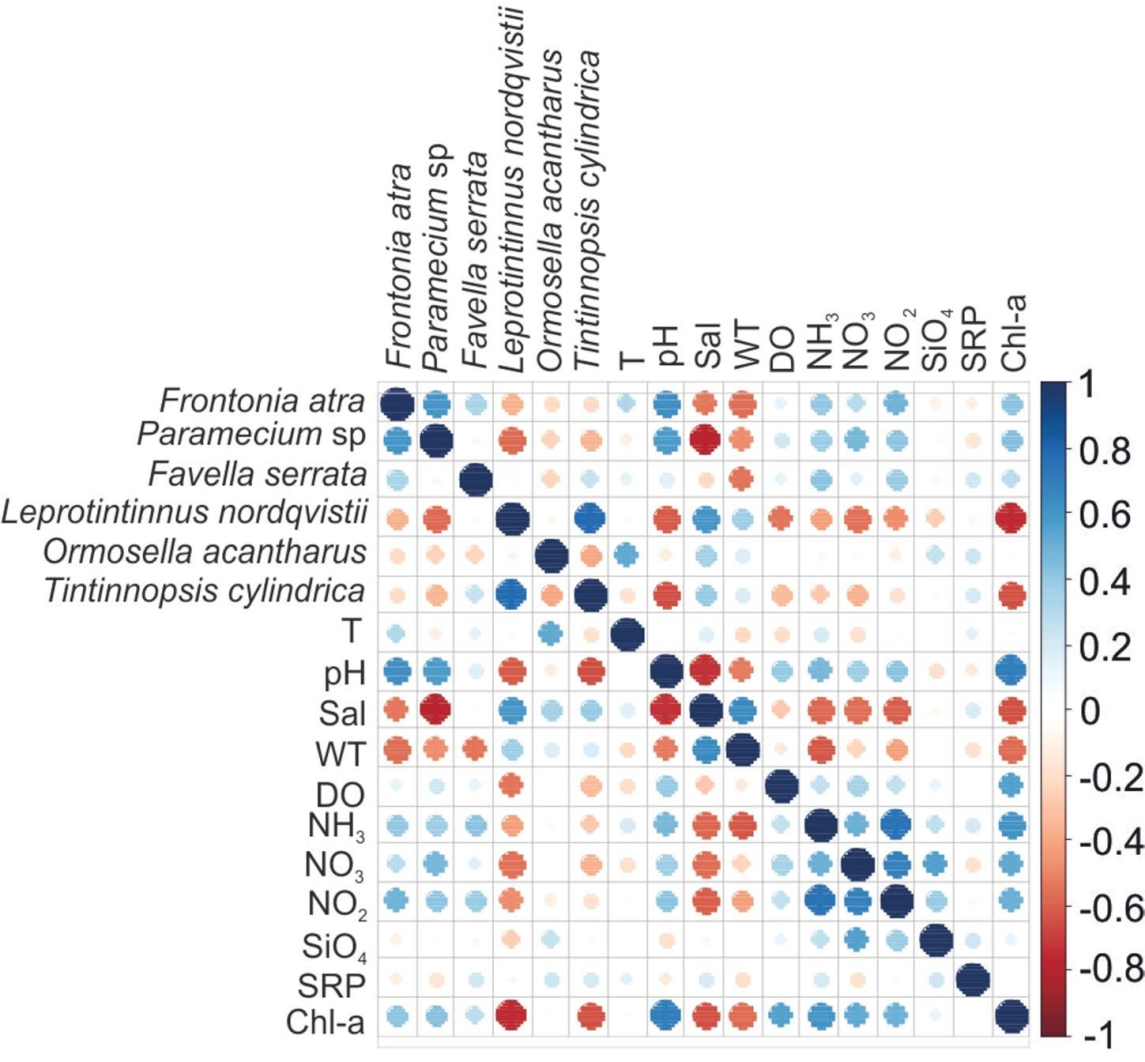
Spearman correlations between environmental parameters and dominant species in the Damietta region. T, temperature; Sal, salinity; WT, water transparency; SRP, soluble reactive phosphate; Chl-a, chlorophyll-a.

For the six dominant planktonic ciliates, all dominant tintinnids except *Favella serrata* showed significant positive correlations with salinity (Fig. 7). The *Leprotintinnus nordqvistii,* and *Tintinnopsis cylindrica* were significantly negatively correlated with pH, ammonia, nitrate, and chlorophyll-a. The aloricate *Frontonia atra* and *Paramecium* sp. were significantly negatively correlated with salinity and water transparency but positively correlated with pH, nitrogenous nutrients, and chlorophyll-a. Other significant positive correlations included those between *Ormosella acantharus* and temperature, *Favella serrata* and ammonia + nitrate, *Leprotintinnus nordqvistii* and transparency. It should be noted that only chlorophyll-a showed a significant negative correlation with species count (r = − 0.352, P ˂ 0.05), Shannon diversity (r = − 0.438, P ˂ 0.01), and species richness (r = − 0.312, P ˂ 0.05), however, all other environmental variables failed to reveal a significant correlation to biodiversity indices.

## Discussion

The multivariate analyses applied in the present study have proved their utility in detecting changes in community structures and evaluating relationships between communities' ecological patterns and environmental parameters along environmental stress gradients. These findings are consistent with many previous studies e.g.,^6,32–34^. The multivariate approaches collectively demonstrated that spatial patterns of planktonic ciliate communities were significantly associated with the spatial changes in environmental status. Furthermore, BIOENV analysis demonstrated that the variations in ciliate community structures were significantly related to specific environmental parameters, especially salinity, either alone or in combination with other factors, particularly chlorophyll-a and temperature. Accordingly, the clear spatial distribution of the ciliate community in the study area was primarily driven by natural environmental forces, mainly salinity, temperature, and trophic status. According to Sanders^35^ and Xu et al.^36^, the ciliate communities are sensitive to changes in environmental variables such as salinity, temperature, nutrient levels, and food supply. These changes could be more pronounced in sheltered systems like estuarine and harbour waters. The study area is an interconnected ecosystem influenced by multiple anthropogenic disturbances^18^. The distribution of hydrographic parameters showed a noticeable variables gradient, revealing the influence of the Nile Delta runoffs and the tidal regime. During the sampling period, the estuarine water intrusion did not significantly affect the CR and DH sites since the salinity obtained here was around 36 ppt, considerably higher than that of the estuarine waters. The high dissolved inorganic nitrogen concentration gradients inside the estuary were due to the land runoff and low water exchange with the adjacent sea^19^. The saltwater intrusion in estuaries is caused by a complex interaction process mainly related to freshwater flux upstream and tidal currents^37^. In the Damietta estuary, the freshwater flow is obstructed by the Farskour dam and the extremely low tidal range at the Nile Delta coast, with a 30-60 cm variation in daily mean sea level^20^, making the land runoffs the key players controlling the water properties in the estuary, particularly upstream.

Our results showed that the abundance of ciliates in the present study was among the highest values ever reported for the Egyptian coastal waters of the Mediterranean Sea (Table 4). Beaver and Crisman^38^ observed great variability in ciliate density depending on the trophic status of the environment. As a rule, the abundance of ciliates increased with eutrophication^39^. The Egyptian coasts, mainly the Nile Delta and Alexandria, were reported to be eutrophic due to very high terrestrial inputs^40^. So, the issue of low ciliate abundances in such eutrophic regions needs to be clarified. The use of different fixatives/preservatives and various methods of sample concentrations can influence estimates of ciliate abundance significantly^41,42^. According to Stoecker et al.^41^, fixation with acid Lugol’s solution, which was used in the present study, results in significantly higher cell counts than fixation with formaldehyde, such as was used as fixative in most other studies in the Egyptian waters. Also, the filtration process could be another reason for the low abundance of ciliates. Sime-Ngando et al.^43^ reported an average loss of 15% of ciliates due to the filtration process, while Lynn et al.^44^, suggest half the community may be smaller than 20 microns in mesotrophic tropical waters. The filtration process with a mesh size bigger than 20 microns applied in other studies (Table 4) could reasonably explain the low ciliate densities reported in Egyptian waters. It’s surprising to some extent that abundances are not higher given the eutrophic status of our region; thus, factors such as predation by mesozooplankton may be controlling populations. Several studies of copepods feeding on natural microplankton assemblages have demonstrated copepods’ predation on both loricate and aloricate ciliates, suggesting ciliates supply from 1 to 80% of total carbon ingested^45,46^. In tropical and subtropical systems, small copepods are often the dominant zooplankton^47,48^ and play a significant role in the cycling of nutrients^49^. The small cyclopoid copepod of the genus *Oithona* is extremely abundant in neritic areas of the tropics and subtropics^50–52^; and in Egyptian waters, *Oithona* is the most abundant copepod genera^18,19,53,54^. Feeding studies on *Oithona* spp. have revealed an omnivorous diet, feeding primarily on ciliates and dinoflagellates^55,56^; phytoplankton, particularly diatoms, have been occasionally reported to make up a considerable fraction of their natural diet^57^. In a mesocosm experiment, Zöllner et al.^58^ reported a drastic decrease in ciliates abundance with increasing copepod abundance and were nearly eliminated with maximal zooplankton densities. So, selective copepod grazing could be responsible for the reduction of ciliates abundance in Egyptian waters. In eutrophic environments, ciliates are thought to be regulated by metazooplankton rather than food supply^59^. Also, coastal hydrodynamics was reported as a limiting factor for the development of ciliates^60^; however, further investigations are needed to clarify this issue. Nonetheless, our abundances fall within the range observed in other studies within oceanic and coastal regions globally (Table 4)^61^, suggesting any biases in our values are not severe.

**Table 4:** Ciliate population densities (cells l^−1^) at different locations.

Location	Abundance (cells l^−1^)	References	Comments
**Egyptian waters**			
Nile Delta	16.5–5575	This study	Abundance range
Damietta Harbour	15–73.5	Dorgham et al.^62^	Vh, 55 µm mesh
Damietta coast	0.5–194.5	Dorgham et al.^10^	F 100 liters, 35 µm mesh
Rosetta estuary	(3.2)	Abo-Taleb^63^	Abundance average—Vh, 55 µm mesh
Western Harbour	1.9–22.7	Heneash et al.^64^	Tintinnids abundance range—Vh, 55 µm mesh
Alexandria coast	446–1262	Galal^65^	1-liter bottle
Damietta Nile estuary	0.47–7.82	El-Tohamy et al.^19^	Vh, 55 µm mesh
Eastern Harbour and El-Max Bay	4.33–21	Sharaf et al.^66^	Total protozoa—F 200 liters, 55 µm mesh
**The Mediterranean Sea**			
Kasˇtela Bay (Adriatic Sea)	0–2500	Bojanic et al.^67^	Abundance range—Nb
Northern Adriatic Sea	0–5490	Monti et al.^68^	Abundance range—Nb
Gulf of Gabes (Tunisia)	300–6300	Elloumi et al.^69^	Abundance range—VDb
The south coast of Sfax (Tunisia)	415–715	Rekik et al.^70^	Average abundance range—VDb
The Bay of Mersin (Turkey)	0–820	Polat et al.^71^	Tintinnids abundance range—F 20 liters, 18 μm mesh
Gulf of Gabes (Tunisia)	1000–16,200	Kmiha-Megdiche et al.^72^	Abundance range at surface layer—Nb
Gulf of Gabes-Boughrara lagoon system (Tunisia)		Rekik et al.^73^	Abundance range—Nb
Coastal waters of Algeria	˂200–716	Ali et al.^74^	Microzooplankton abundance range—Nb
**Other areas**			
Lime Cay, Jamaica	1000–3900	Lynn et al.^44^	aloricate spp. only—Nb
Lime Cay, Jamaica	0.74–340	Gilron et al.^75^	Tintinnids abundance range—Vh, 20 µm mesh
Northwestern Indian Ocean:	31–823	Leakey et al.^76^	Abundance range—10 liters Go-Flo bottles
Gulf of Maine	400–6000	Montagnes et al.^77^	2-liter bottle
East China Sea	3–2688	Yang et al.^78^	Abundance range—Nb
South China Sea	200–˂ 3000	Liu et al.^79^	Abundance range
Northern South China Sea (Subtropical Pearl River estuary)	7.5–3900	Gu et al.^80^	Abundance range—Nb

Sampling methods abbreviations: VDb, Van Dorm bottle; Nb, Niskin bottle; F, Filtration; Vh, Vertical hauls.

The lowest ciliate numbers in the estuary downstream and the highest ciliate numbers in the estuary upstream fall together with the highest values of nutrients and chlorophyll-a. The data correlating trophic state to the ciliate abundance contribute to that hypothesis. For the estuary upstream, a significant increase in ciliate densities with increasing trophic state was found, corresponding to results from several studies e.g.,^9,81–84^. However, the significant decrease in ciliate abundance downstream may be due to the toxic effects of antifouling paint particles on the survival and growth of protozoa species^85^. Antifouling paints comprise various substances, including pigments, metals, and hydrocarbons, major chemical contaminants in estuaries^86,87^. When present in high concentrations, they are possibly damaging the estuarine organisms. The downstream region hosts about 65% of the Egypti an fisheries fleet^88^; antifouling paint particles are usually released into waters during in-water cleaning activities to manage ships' hulls for repair, cleaning, and painting.

The regulation of ciliates is influenced by the biomass of phytoplankton, bacterioplankton, and heterotrophic nanoflagellates^67^. Many ecological models used phytoplankton biomass as a common denominator for predicting ciliate abundance; they considered it a measurable food source for algivores ciliates^89^. Ciliates can tightly regulate phytoplankton grazing, consuming between 60 and 70% of the sea’s total primary productivity^90^. In the Egyptian coastal water, photosynthetic pico- (0.2–2 μm) and nanoplankton (2–20 μm) comprise the majority of phytoplankton biomass^91,92^. In the York River estuary, The contribution of large cells (microplankton, > 20 μm) to total phytoplankton biomass increased during winter, whereas that of small cells (pico- and nanoplankton) increased during summer^93^. Unfortunately, we don’t have any previous studies about pico- and nanoplankton in the study area; the present study sampling was during summer, which may reflect the effective contribution of small cells to the phytoplankton biomass, particularly in the Nile estuary and BC. According to Rassoulzadegan et al.^94^, a significant biomass of pico and nanoplankton in aquatic systems is believed to be heterotrophic. Inorganic nutrients and light can influence both the autotrophic and heterotrophic states^95^. Globally, coastal zones are heterotrophic^96^; rates of heterotrophic activity can exceed primary production in many aquatic ecosystems^95^. A significant input of nutrients in the study area with sufficient summer light is undoubtedly important in moments of increased autotrophic and heterotrophic growth. In the present study, we found no significant correlation between the total ciliate abundance and chlorophyll-a (r = − 0.233, p = 0.104), although the PCA suggests a negative link in the harbour and coastal region where tintinnids were the dominant and a positive one at the estuary that dominated by aloricate ciliates. Also, correlation analysis suggests a positive association between chlorophyll-a and aloricate ciliates (r = 0.560, p ˂ 0.001) and a negative association with tintinnids (r = − 0.448, p ˂ 0.01). We have to admit here that autotrophic organisms are not the only denominators of ciliate food sources, as indicated by the results of statistical analyses. The positive relationship of aloricate ciliates with phytoplankton biomass indicates possible trophic relationships consistently with the dominance of the autotrophic cells in the Nile estuary and BC, particularly the genera *Frontonia* and *Paramecium*, which represented the most numerous trophic assemblage in the estuary^19^. In contrast, the tintinnid ciliates appeared not to be controlled by the availability of autotrophic prey, as suggested by the absence of a relationship between them and the phytoplankton biomass in the present study. Our data confirmed previous results of Sitran et al.^97^ that tintinnid diversity and abundance were negatively correlated to chlorophyll-a in the water column. Tintinnids have been shown to exhibit wide food plasticity (detritus, picoplankton, bacterioplankton, and nanoflagellates)^61,73,97^ in different ecosystem conditions, which may explain the lack of a clear link between phytoplankton biomass and tintinnid abundance or diversity. With the decreasing micro phytoplankton biomass, tintinnids are generally unable to feed on colonial diatoms or consume large prey^97^. Tintinnids have shifted their feeding toward bacterioplankton and nanoflagellates during the decline of phytoplankton biomass in the harbour and coastal regions. Din^98^ repoted a significant decrease in nanoflagellates biomass in parallel with the tintinnid increased biomass.

Species distribution of 32 ciliate species, particularly the six dominant species, represented a clear spatial pattern that contributed to the spatial variations in the ciliate communities within the study area. These dominant taxa belong to spirotrichs (e.g., *Tintinnopsis* and *Favella*) and oligohymenophoreans (e.g. the scuticociliates *Paramecium*), commonly encountered in freshwater and marine habitats. The ciliates can tolerate extreme salinity changes, and some can sustain direct transfer from marine to freshwater^99^. Therefore, ciliate communities in estuarian and coastal marine environments probably include freshwater, brackish, and marine species^100^. To date, the only available detailed studies about the ciliate diversity in the region were from the Damietta Harbour, with 37 species (Dorgham et al.^62^, the Damietta coastal region, with 49 species^10^, and the Nile estuary of Damietta Branch with 22 species,^19^. These differences in ciliate diversity are likely due to the study sites, the climate variability, the sampling period/and or sampling protocols applied in the different studies^74^. The dominance of the tintinnid ciliates during the present investigation is consistent research performed on a cross-wide range of marine trophic levels^70,72^. The strong presence of tintinnid in all domains except the upstream estuary might be coupled with the high salinity values and a better adaptation of many tintinnids to lower nutrient concentrations. Aloricate ciliates usually dominate the ciliate community in the upstream region^19^ since they prefer conditions of low salinity and higher nutrient concentrations. According to Martínez-López et al.^101^, the decrease in tintinnid abundance is associated with increased gradients of environmental stress that could be more favourable to aloricate ciliates.

Species diversity, evenness, and richness indices are commonly used to summarize the community biodiversity^102^ and are usually utilized as amenable indicators for assessing water quality^5,6,8,32,33^. In general, the higher values of these three indices usually reflect better water conditions^6,8^. In the present study, the three biodiversity indices employed showed relatively higher values in the samples from less stressed domains (e.g. the coastal region) than in the samples from more stressed ones (e.g. the estuary upstream). According to Huston^103^, the changes in diversity can only be assessed when sites are compared along spatial contamination gradients. Wong and Dowd^104^ successfully applied diversity indices to assess environmental changes across anthropogenic impacts. The selected regions in our study are usually characterized by natural gradients linked to the sites-sea or sites-lake (Manzala) interface that are anthropized by chemical contaminants, potentially affecting the diversity of ciliate communities. Acute and permanent anthropogenic stress in the estuary upstream may hinder adaptation by rare species, and potentially eliminating them from the species pool lowered the diversity of ciliate communities^105,106^. In contrast, according to Xu et al.^107^, planktonic ciliates ecological response to the influence of eutrophication can be complex and non-linear. In the present study, we found that nutrients had no direct impact on ciliate diversity indices; however, chlorophyll-a was inversely correlated, based on the Spearman correlation analysis. It is worth mentioning that the diversity indices are influenced by species dominance, which may be controlled by environmental variables unrelated to pollution, such as salinity and food availability.

## Conclusion

The planktonic ciliate community structures represented significant differences among the sampling domains. The spatial variations in the community structural parameters were significantly related to the spatial changes of environmental variables, especially salinity, either alone or in combination with other parameters. The distribution patterns of tintinnid and aloricate ciliates between the sampling precisely reflect the gradients of ecological parameters and are a significant part of assessing the environmental status. Six species dominated the 32 ciliate taxa recorded over the study period, significantly related to salinity and/or nutrients or chlorophyll-a. Our results suggest that the spatial pattern of planktonic ciliate communities and taxonomic biodiversity could be used as favourable bioindicators for monitoring water quality status for the management of this region. With their short life cycle, delicate pellicle, and easy sampling and identifying features, these planktonic ciliates have specific advantages to be used to assess environmental changes in aquatic ecosystems compared to metazoans. However, further studies over long-term periods are needed to verify this conclusion.

## Data Availability

The datasets used and analyzed during the current study are available from the corresponding author upon reasonable request.
